# Acute Effects of Glucagon on Reproductive Hormone Secretion in Healthy Men

**DOI:** 10.1210/clinem/dgaa164

**Published:** 2020-03-31

**Authors:** Chioma Izzi-Engbeaya, Sophie Jones, Yoshibye Crustna, Pratibha C Machenahalli, Deborah Papadopoulou, Manish Modi, Jessica Starikova, Derek Chan, Pei Chia Eng, Maria Phylactou, Risheka Ratnasabapathy, Edouard Mills, Lisa Yang, Ewa Pacuszka, Paul Bech, James Minnion, George Tharakan, Tricia Tan, Johannes Veldhuis, Ali Abbara, Alexander N Comninos, Waljit S Dhillo

**Affiliations:** 1 Section of Endocrinology and Investigative Medicine, Imperial College London, London, UK; 2 Department of Endocrinology, Imperial College Healthcare NHS Trust, London, UK; 3 Department of Acute Medicine, Imperial College Healthcare NHS Trust, London, UK; 4 Department of Internal Medicine, Mayo Clinic, Rochester, Minnesota

**Keywords:** glucagon, luteinizing hormone, follicle-stimulating hormone, testosterone, reproduction

## Abstract

**Context:**

Glucagon increases energy expenditure; consequently, glucagon receptor agonists are in development for the treatment of obesity. Obesity negatively affects the reproductive axis, and hypogonadism itself can exacerbate weight gain. Therefore, knowledge of the effects of glucagon receptor agonism on reproductive hormones is important for developing therapeutics for obesity; but reports in the literature about the effects of glucagon receptor agonism on the reproductive axis are conflicting.

**Objective:**

The objective of this work is to investigate the effect of glucagon administration on reproductive hormone secretion in healthy young men.

**Design:**

A single-blinded, randomized, placebo-controlled crossover study was conducted.

**Setting:**

The setting of this study was the Clinical Research Facility, Imperial College Healthcare NHS Trust.

**Participants:**

Eighteen healthy eugonadal men (mean ± SEM: age 25.1 ± 1.0 years; body mass index 22.5 ± 0.4 kg/m^2^; testosterone 21.2 ± 1.2 nmol/L) participated in this study.

**Intervention:**

An 8-hour intravenous infusion of 2 pmol/kg/min glucagon or rate-matched vehicle infusion was administered.

**Main Outcome Measures:**

Luteinizing hormone (LH) pulsatility; LH, follicle-stimulating hormone (FSH), and testosterone levels were measured.

**Results:**

Although glucagon administration induced metabolic effects (insulin area under the curve: vehicle 1065 ± 292 min.µU/mL vs glucagon 2098 ± 358 min.µU/mL, *P* < .001), it did not affect LH pulsatility (number of LH pulses/500 min: vehicle 4.7 ± 0.4, glucagon 4.2 ± 0.4, *P* = .22). Additionally, there were no significant differences in circulating LH, FSH, or testosterone levels during glucagon administration compared with vehicle administration.

**Conclusions:**

Acute administration of a metabolically active dose of glucagon does not alter reproductive hormone secretion in healthy men. These data are important for the continued development of glucagon-based treatments for obesity.

The global increase in the prevalence of obesity has fuelled the search for effective and safe treatments for obesity and related disorders. Glucagon is produced by pancreatic α-cells and increases energy expenditure, satiety, hepatic glucose output, and insulin secretion (1). Glucagon receptor agonists (together with other gut hormone receptor agonists) have been shown to reduce body weight and improve glucose tolerance in rodents and humans (2-6). Because the metabolic effects of glucagon receptor agonists are being used in the development of novel antiobesity treatments, it is important to determine whether there are additional direct effects of glucagon receptor agonism on reproductive hormone secretion aside from those due to changes in body weight. Furthermore, this information would improve our understanding of the dynamic metabolism-reproductive interface and help determine whether the impact of obesity on the endocrine reproductive axis could be in part mediated through glucagon.

Low reproductive hormone levels (known as male obesity secondary hypogonadism, MOSH) occurs in 25% to 40% of men with obesity (7). Consequently, if glucagon receptor agonism has a direct effect on the reproductive system (in addition to its metabolic effects), this would be highly clinically relevant because hypogonadism worsens insulin resistance, facilitates weight gain, impairs fertility, and worsens quality of life (7). Although some evidence (detailed as follows) suggests glucagon may influence reproductive hormone secretion, the nature of its effects on reproductive hormones has not been fully characterized. Additionally, no information about reproductive hormone levels were provided in studies exploring the metabolic effects of glucagon receptor (co-)agonists (2-6).

A study in ovariectomized estrogen-primed female rats demonstrated that 1 µg of glucagon injected into the medial preoptic area of the hypothalamus increased peak serum luteinizing hormone (LH) levels 4 hours postinjection (8). In humans, a 1 mg dose of glucagon given intravenously did not affect LH levels in the limited time frame studied (ie, 180 minutes) (9). In contrast, 0.03 mg/kg glucagon injected intramuscularly resulted in significant increases in serum LH in 11 of 13 people (peaking at 2 hours after glucagon administration), but again the effects of glucagon administration were measured for only 180 minutes (10).

Because glucagon receptor agonists are being developed to treat obesity (2-6), which is frequently complicated by MOSH (7), it is important to conclusively determine whether glucagon receptor agonism can directly modulate the reproductive axis in men. Based on animal data, we hypothesized that glucagon could directly stimulate the reproductive axis; however, the evidence base in humans remains uncertain. Therefore, we conducted a single-blinded, placebo-controlled study to determine the direct effects of acute glucagon administration on the reproductive axis in men.

## Methods

### Participants

This study was performed in accordance with the Declaration of Helsinki and received ethical approval from the West London Research Ethics Committee (16/LO/0391) and was registered on the ISRCTN clinical trial registry (ISRCTN10114288). Following recruitment using online and print advertisements, screening, and informed consent, 18 healthy men (mean age 25.1 ± 1.0 years; mean body mass index [BMI] 22.5 ± 0.4 kg/m^2^; testosterone 21.2 ± 1.2 nmol/L) were entered into the study. Exclusion criteria included: BMI less than 18.5 or greater than 25 kg/m^2^, history of medical and psychological conditions, use of prescription, recreational or investigational drugs within the preceding 2 months, blood donation within 3 months of study participation, ingestion or inhalation of nicotine-containing substances within 3 months, alcoholism, or history of cancer.

### Study visits

Each participant attended 2 study visits, 1 for glucagon administration and 1 for vehicle administration. The order of the infusions was randomized (using www.random.org) and participants were blinded as to the identity of the infusions. Glucagon infusions were prepared by dissolving 1 mg glucagon in 1 mL of sterile water and adding the glucagon solution to 49 mL Gelofusine (Braun). Glucagon was administered at a rate of 2 pmol/kg/min, a dose previously established to be metabolically active in humans (11). Rate-matched vehicle infusions comprised Gelofusine (Braun) only.

Participants fasted from 10 pm the night preceding each study visit. On the morning of each study visit, participants ate a standardized 200 kcal breakfast (OatSoSimple, Quaker Oats Ltd) at 6 am and arrived at the Clinical Research Facility at 8:15 am. After a period of acclimatization, 2 intravenous cannulae (1 in each arm) were inserted (1 cannula was used for blood sampling and the second for administering the infusion). Following baseline sampling, a glucagon/vehicle infusion was started at T = 0 min and continued until T = 500 min. A visual analog scale (0-10 cm), was used to measure participants’ self-reported nausea at T = –15 min, T = 240 min, and T = 470 min. Participants were given an ad libitum meal at T = 480 min. Blood samples were taken every 10 minutes throughout the study for assessment of serum insulin, LH, follicle-stimulating hormone (FSH), and testosterone (apart from during the meal).

### Biochemical analyses

Serum insulin, LH, FSH, and testosterone were measured by North West London Pathology on the automated Abbott Architect platform. Chemiluminescent immunoassays were used to measure serum insulin (intra-assay and interassay coefficient of variation [CV]: ≤ 7%), serum LH (intra-assay and interassay CV: ≤ 5%), serum FSH (intra-assay and interassay CV: ≤ 10%), and serum testosterone (intra-assay and interassay CV: ≤ 8%). Blood glucose was measured using a FreeStyle Optimum Meter (Abbott Diabetes Care). Plasma glucagon was measured using a well-established in-house radioimmunoassay (intra-assay and interassay CV: ≤ 10%) (12).

### Main outcome measures

The primary outcome measures were LH levels and LH pulsatility. Secondary outcomes included FSH and testosterone levels.

### Statistical methods

The study’s sample size (n = 18 per group) was chosen to provide 90% power to detect a difference in LH (between vehicle and glucagon administration) of 2 IU/L (SD 2.3 IU/L) at a significance level of .05 (13). LH pulsatility was assessed by J.V. (who was blinded to the identity and order of the intravenous infusions) using a validated deconvolution analysis (ie, a mathematical model of the underlying secretion and/or elimination rates of LH, derived from the LH concentration profile, which identifies and characterizes LH pulses and pulse orderliness [regularity]) (14, 15). Pulse orderliness refers to the regularity of pulsatile hormone secretion. In our study, LH pulse orderliness was determined by calculation of the approximate entropy (ApEn) of the profile of LH secretion as part of the deconvolution analysis. When LH pulsatile secretion is less orderly, it is more irregular and has a higher ApEn.

Longitudinal data were analyzed using generalized estimating equations with STATA 15.1 software (STATA Corp). Paired t tests were performed on parametric data and Wilcoxon matched-pairs signed rank tests were performed on nonparametric data using Prism 8.0.2 (GraphPad) software. *P* values of less than .05 were considered statistically significant. Data are presented as mean ± SEM.

## Results

### Confirmation of biological activity of glucagon administration

**Figure 1.**
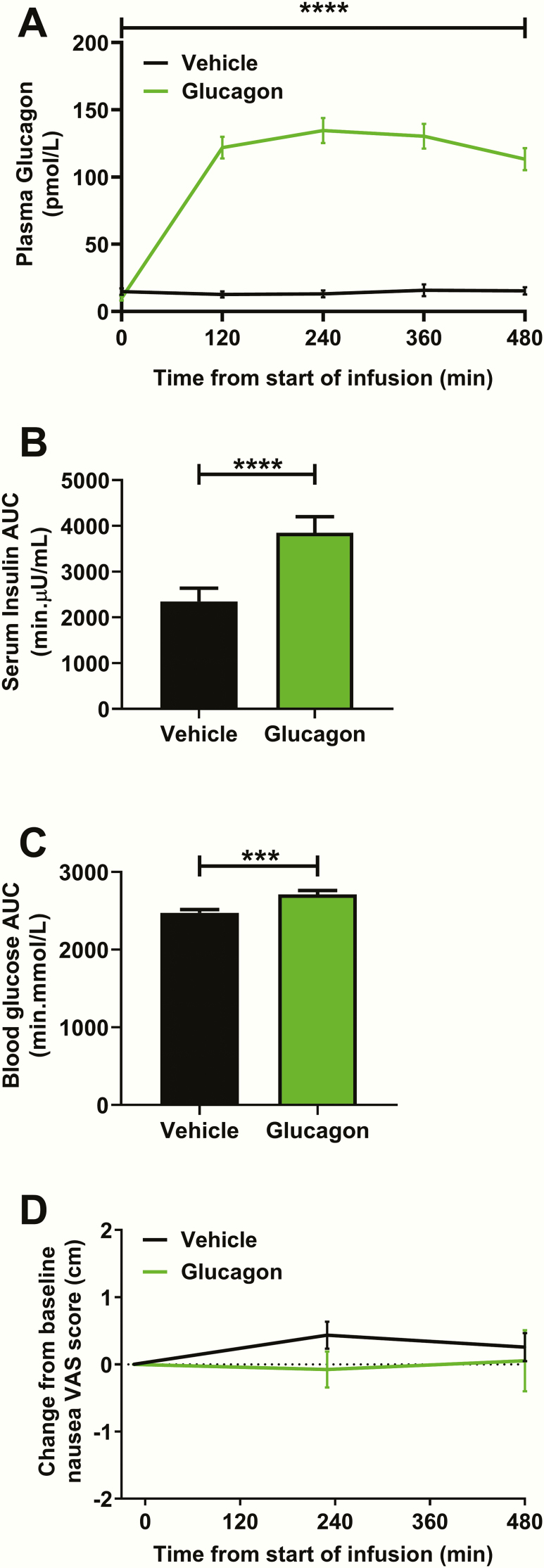
Confirmation of biological activity of administered glucagon. A, Glucagon levels were significantly higher during glucagon administration compared to during vehicle administration (vehicle vs glucagon from T = 0 min to T = 480 min, ****P < .001 using generalized estimating equation [GEE]). B, Insulin area under the curve (AUC) was significantly higher during glucagon administration compared to vehicle administration (****P < .001 using a Wilcoxon matched-pairs signed rank test). C, Glucose AUC was significantly higher during glucagon administration compared to vehicle administration (***P < .001 using a paired t test). D, There was no significant difference in self-reported (change from baseline) nausea during glucagon administration compared with vehicle administration (vehicle vs glucagon from T = –15 min to T = 470 min, *P* = .32 using GEE). Abbreviation: VAS, visual analog scale (0-10 cm).

Intravenous infusion of glucagon at 2 pmol/kg/min was confirmed to significantly increase circulating plasma glucagon levels (Fig. 1A). Preinfusion insulin (vehicle 7.8 ± 1.0 µU/mL vs glucagon 10.4 ± 3.4 µU/mL, *P* > .99) and glucose (vehicle 5.1 ± 0.2 mmol/L vs glucagon 5.2 ± 0.2 mmol/L, *P* = .26) were similar in both groups. Glucagon administration resulted in a higher insulin area under the curve (AUC) (Fig. 1B) and glucose AUC compared to vehicle administration (Fig. 1C). Glucagon administration is recognized to cause nausea (9), which (as a form of stress) could have had a detrimental impact on reproductive hormone release (16). However, the dose of glucagon used in the present study although metabolically active (as shown earlier), did not cause nausea (Fig. 1D) and did not affect food intake (kcal eaten/kg body weight: vehicle 15.5 ± 0.3 vs glucagon 17.0 ± 1.3, *P* = .11).

### Effects of glucagon administration on reproductive hormones

**Figure 2.**
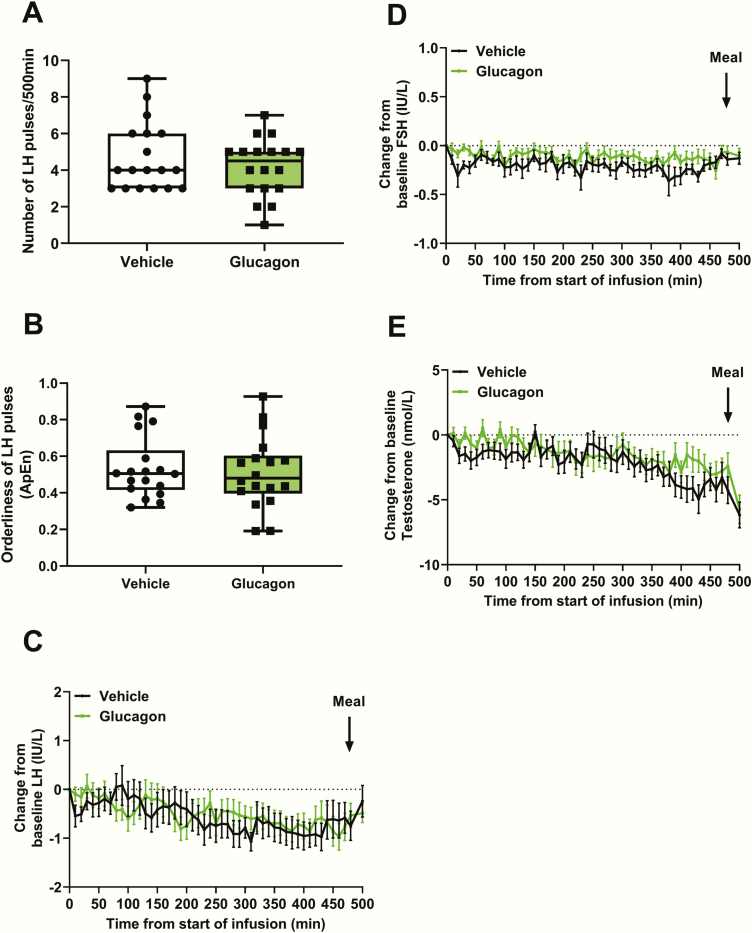
Effects of glucagon administration on reproductive hormone levels. A, The number of luteinizing hormone (LH) pulses was similar during glucagon and vehicle infusions (*P* = .22 using a Wilcoxon matched-pairs signed rank test). B, LH pulse orderliness (ie, approximate entropy [ApEn]—with zero representing perfect orderliness) was similar during glucagon and vehicle infusions (*P* = 0.73 using a Wilcoxon matched-pairs signed rank test). C, Change from baseline LH was similar during 2 pmol/kg/min intravenous glucagon infusion and rate-matched vehicle infusion (vehicle vs glucagon from T = 0 min to T = 500 min, *P* = .83 using generalized estimating equation [GEE]). Meal = ad libitum meal. D, Change from baseline follicle-stimulating hormone (FSH) was similar during 2 pmol/kg/min intravenous glucagon infusion and rate-matched vehicle infusion (vehicle vs glucagon from T = 0 min to T = 500 min, *P* = .11 using GEE). Meal = ad libitum meal. E, Change from baseline testosterone was similar during 2 pmol/kg/min intravenous glucagon infusion and rate-matched vehicle infusion (vehicle vs glucagon from T = 0 min to T = 500 min, *P* = .36 using GEE). Meal = ad libitum meal.

Glucagon administration did not affect the number of LH pulses (number of LH pulses/500 min: vehicle 4.7 ± 0.4 vs glucagon 4.2 ± 0.4, *P* = .22) (Fig. 2A), nor did it affect the orderliness (ie, regularity) of the LH pulses as measured by LH pulse ApEn (LH ApEn: vehicle 0.53 ± 0.04 vs glucagon 0.51 ± 0.05, *P* = .73) (Fig. 2B). Furthermore, starting from similar baseline levels (mean baseline LH: vehicle 3.6 ± 0.3 IU/L vs glucagon 3.6 ± 0.3 IU/L, *P* > .99), there was no significant change from baseline LH during glucagon administration compared to vehicle administration (Fig. 2C).

Starting from comparable baseline levels (mean baseline FSH: vehicle 2.7 ± 0.3 IU/L vs glucagon 2.8 ± 0.3 IU/L, *P* = .22), the change from baseline FSH was similar during glucagon administration compared to vehicle administration (Fig. 2D). Likewise, testosterone levels at baseline were similar (mean baseline testosterone: vehicle 23.3 ± 1.7 nmol/L vs glucagon 23.4 ± 1.7 nmol/L, *P* = .93), and there was no significant difference in the change in testosterone from baseline during glucagon and vehicle administration (Fig. 2E).

## Discussion

We demonstrate that acute glucagon administration, at a dose that produces metabolic effects (as evidenced by higher insulin and glucose AUC compared to vehicle administration), does not affect the number of LH pulses nor LH pulse orderliness; nor does it affect LH, FSH, or testosterone levels. Previous human studies have suggested that glucagon administration either had no effects on reproductive hormone release (9) or had stimulatory effects on LH (10). However, these studies were performed on a small number of male volunteers with blood samples taken for a relatively short period of time, and testosterone levels were not reported in these studies (9, 10). Additionally, normal reproductive function is dependent on LH pulsatility as well as absolute LH levels (17), and LH pulsatility was not assessed in these previous studies (9, 10). Furthermore, the previous studies were performed more than 40 years ago, and differences in historical hormone measurement compared with modern techniques (including assay sensitivity and specificity) may limit interpretation of these previous studies’ results. Therefore, firm conclusions could not be drawn from the existing literature.

By comparison, the present study is the first to extensively characterize the effect of acute glucagon administration on reproductive hormone release in men using validated methodology (blinded deconvolution analysis of LH pulsatility) for an extended period of time (ie, 500 minutes) using a protocol designed to detect clinically relevant differences while avoiding potential confounding factors. The study duration was chosen to ensure sufficient numbers of LH pulses occurred for adequate pulse analysis because the time between LH pulses in healthy men has been shown to range from 15 minutes to more than 135 minutes, with a mean interpulse interval of 111minutes when samples are taken every 10 minutes (18). Therefore, it would be expected that on average 4 LH pulses would occur during the 500-minute period during which glucagon was administered in our study; and our protocol (ie, blood samples taken every 10 minutes) ensured the LH pulses that occurred would be detected. Fasting and eating both can affect reproductive hormone levels, thus we designed the study to avoid both extremes of prandial status. Fasting suppresses LH release and pulsatility (13), and conversely testosterone levels are suppressed in the immediate postprandial period (19). Therefore, study participants consumed a meal at 6 am before the start of the glucagon infusion to minimize the confounding effects of prandial status on reproductive hormone secretion.

It would be interesting to examine the effects of chronic glucagon receptor agonist administration on reproductive hormones in the future because there may be differences between acute effects (as reported in this study) and chronic effects of glucagon receptor agonism, and currently there are no reports of the effects of recently developed agonists on reproductive hormone secretion (2-6). However, interpretation of the direct effects of glucagon agonism on reproductive hormones using these agents will be challenging because the weight loss associated with these agents would be likely to improve hypogonadism if present.

A possible explanation for the absence of an effect of glucagon administration on reproductive hormone levels is nausea, which can be caused by glucagon receptor agonism (9). Nausea (as a form of stress) may have a detrimental impact on reproductive hormone release (16). However, the dose of glucagon used in this study produced some of the expected metabolic effects (ie, an increase in glucose and insulin concentrations) but did not increase nausea (compared to vehicle administration). In contrast to some of the literature, the dose of glucagon administered in our study did not reduce food intake. This may be due to differences in study methodology because, in our study, food intake was assessed more than 8 hours after previous nutrient ingestion, whereas in other studies reporting a reduction in food intake with glucagon administration, food intake was assessed 20 to 210 minutes after previous nutrient ingestion (20, 21).

Another plausible explanation for our findings is that peripherally administered glucagon was unable to act centrally and therefore could not influence hypothalamic function. However, there is evidence to suggest that intravenously administered glucagon crosses the blood-brain barrier (22). Therefore, it is reasonable to conclude that central glucagon receptor agonism is also not likely to result in acute changes in reproductive hormone secretion.

Our finding that glucagon administration does not affect reproductive hormones in healthy men provides important data on potential off-target effects in the ongoing development of glucagon receptor agonists for the treatment of obesity. Based on our data, therapeutic glucagon receptor agonists are likely to produce metabolic effects without having adverse direct effects on the reproductive system. Furthermore, chronic exposure to glucagon receptor agonists may improve the reproductive hormone status of men with obesity-related hypogonadism because weight loss itself can ameliorate hypothalamic hypogonadism in obese men (7).

Novel glucagon/glucagon-like peptide-1 (GLP-1) receptor dual agonists and glucagon/GLP-1/glucose-dependent insulinotropic peptide (GIP) receptor triagonists are currently being developed to treat obesity and associated comorbidities (2-6). However, the effects of these agents on reproductive hormone secretion has not been reported (2-6, 23), but they are unlikely to have direct effects on reproductive hormones based on studies using individual constituent hormone receptor agonists. Acute administration of GLP-1 does not alter reproductive hormone levels in healthy men (24). Chronic treatment with GLP-1 receptor agonists has been reported to improve reproductive hormone levels in hypogonadal men with obesity and/or type 2 diabetes, but this may be a consequence of the weight loss associated with GLP-1 receptor agonists (25, 26). In rodents, incubation of pituitary cells with GIP resulted in LH and FSH release, whereas central administration of GIP to female rats lowered circulating FSH levels but had no effect on circulating LH levels (27).

In conclusion, this comprehensive study demonstrates that in healthy men, acute glucagon administration (at a dose that produces metabolic effects) does not affect LH pulse number, or LH pulse orderliness, nor does it affect circulating LH, FSH, and testosterone levels. This provides important mechanistic insight into the direct effects of glucagon receptor agonism on the male reproductive axis. Furthermore, it remains possible that a stimulatory effect of glucagon agonism could be observed in obese men with MOSH with low baseline LH levels and/or pulsatility (28). Consequently, it would be important to now perform similar studies in patient groups such as obese men with MOSH.

## Abbreviations

ApEn: approximate entropy
BMI: body mass index
GIP: glucose-dependent insulinotropic peptide
GLP-1: glucagon-like peptide-1
LH: luteinizing hormone
MOSH: male obesity secondary hypogonadism

## Additional Information

***Disclosure Summary:*** The authors have nothing to disclose.

***Data Availability:*** Restrictions apply to the availability of data generated or analyzed during this study to preserve patient confidentiality or because they were used under license. The corresponding author will on request detail the restrictions and any conditions under which access to some data may be provided.
